# Energy’s long game: thermogenic adipocytes and the metabolic diseases of modern life

**DOI:** 10.20935/acadbiol8131

**Published:** 2026-03-03

**Authors:** Silvia Corvera, Michael P. Czech

**Affiliations:** 1 Program in Molecular Medicine, UMass Chan Medical School, Worcester, MA, USA.

**Keywords:** adipose tissue metabolism, thermogenic adipose tissue, energy homeostasis, mitochondrial uncoupling (UCP1), cardiometabolic disease, evolution of energy metabolism, aging and metabolic regulation

## Abstract

The ability to generate, store, and mobilize energy is fundamental to life, and disruptions in these processes underlie metabolic disease. This review examines how these mechanisms evolved to rely profoundly on lipids, a large family of molecules that serve an unusually wide range of biological functions. From early evolutionary times, lipids have enabled chemiosmotic energy transduction, organized the endomembrane systems that synthesize and package neutral lipids, served as concentrated reservoirs of metabolic energy, and functioned as signaling molecules that coordinate cellular and systemic physiology. Adipocytes are specialized cells that can accumulate large amounts of lipid within specialized lipid droplet organelles. Over evolutionary time, mammals developed distinct adipocyte subtypes with tailored physiological roles. White adipocytes, characterized by a single large unilocular lipid droplet, coordinate energy storage and release in response to systemic cues. In contrast, the role of brown and beige adipocytes is to protect the organism from cold exposure by generating heat as a primary output of UCP1-mediated mitochondrial uncoupling. Crucially, this thermogenic process requires extensive systemic coordination: sympathetic neural input triggers lipolysis, vascular networks deliver fuel from multiple adipose depots, and hormonal signals integrate metabolic demand across organs. These requirements position thermogenic adipocytes as metabolic integration “nodes” that orchestrate whole-body fuel allocation and energy homeostasis. The presence of functional thermogenic adipocytes is strongly associated with improved cardiometabolic health, protecting against obesity, type 2 diabetes, and cardiovascular disease. Understanding how these specialized cells sense and respond to systemic signals offers a powerful entry point for developing strategies to counteract metabolic disease.

## 1. Introduction

The ability to store energy efficiently and mobilize it on demand has shaped the evolution of complex life. In vertebrates, adipose tissue emerged as the central organ system for managing long-term lipid reserves, buffering nutrient flux, and communicating metabolic status to other tissues. Over time, adipocytes diversified into specialized subtypes that support not only energy storage but also endocrine, immune, and vascular functions [1, 2].

Among these specializations, thermogenic adipocytes represent a major evolutionary innovation. Their capacity to convert lipids directly into heat through mitochondrial uncoupling, and the systemic adaptations required to sustain this process, suggest a role that extends well beyond thermal defense.

The following sections examine how energy-storage strategies emerged across evolution, how adipocytes and adipose depots diversified, and how thermogenic adipocytes came to occupy a central position in whole-body metabolic regulation.

## 2. Evolution of energy generation and storage

**Figure 1 •:**
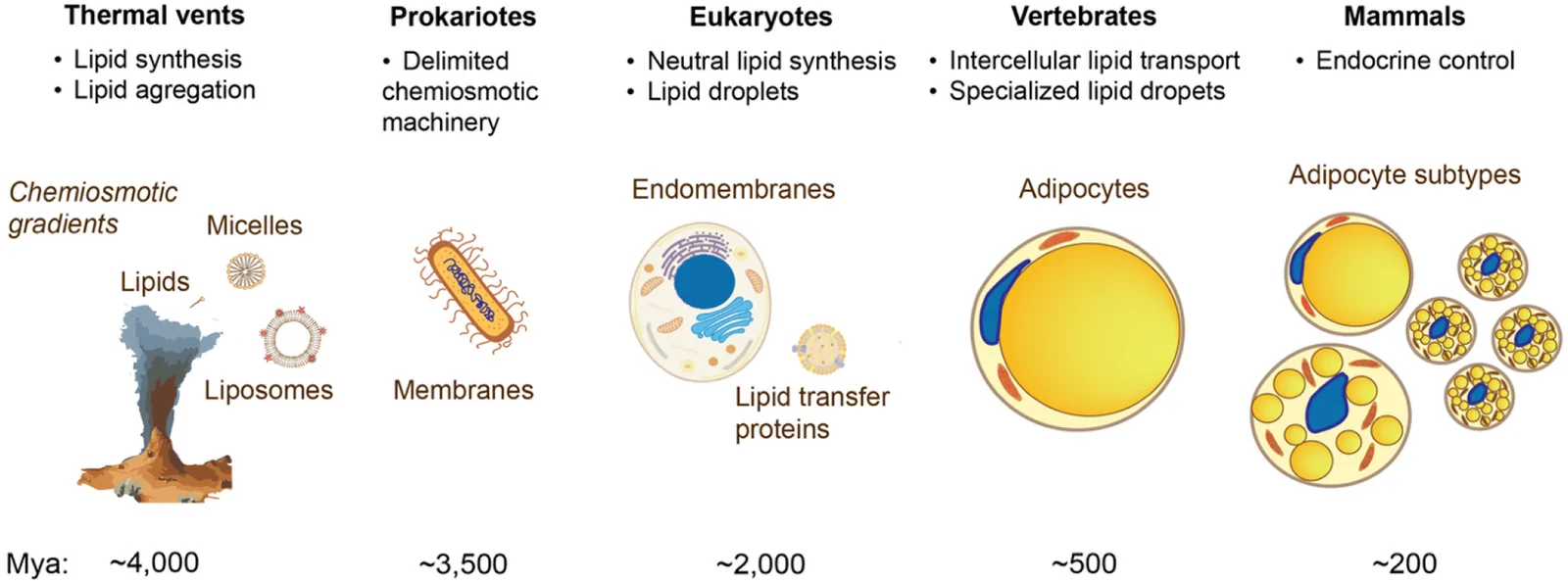
Evolutionary emergence of lipid-based energy storage systems. Early geochemical contexts, exemplified here by thermal vent environments, supported lipid synthesis, aggregation, and the establishment of chemiosmotic gradients through spontaneous self-assembly of amphiphilic molecules. The emergence of prokaryotes introduced membrane-delimited chemiosmotic machinery, allowing energy capture to be spatially constrained within cells. Eukaryotes evolved endomembrane systems that enabled neutral lipid synthesis, formation of lipid droplets, and intracellular lipid trafficking. In vertebrates, the appearance of dedicated adipocytes and intercellular lipid transport mechanisms supported tissue-level and organism-wide energy distribution. Mammals further refined these systems through the emergence of specialized adipocyte subtypes and endocrine regulation, allowing adipose tissue to function as an integrated metabolic and signaling organ. Approximate evolutionary time points (millions of years ago, Mya) are indicated to provide context and are not intended to imply a strictly linear or exclusive evolutionary trajectory.

Life originated ~4 billion years ago under circumstances that fortuitously resulted in the establishment of natural electrochemical gradients, thus capturing energy that supports the anti-entropic arrangement of chemical reactions over a sustained period of time (Figure 1). At any scale—be it a single cell, an organism, or even an ecosystem—life maintains pockets of order in an entropic universe through controlled chemistry [1, 2].

These ancient geochemical gradients provided energy to drive ATP synthesis, a universal feature of all life. Over eons of evolutionary pressure, these systems became more diverse and efficient, with the development of specialized organelles such as mitochondria and chloroplasts for efficient ATP production, and, critically, long-term energy storage systems, including polysaccharides (glycogen and starch) and lipids. Lipids provide ~9 kcal/g vs. ~4 kcal/g for carbohydrates, becoming the favored form of long-term energy storage in most organisms, capable of storing energy for days, weeks, or longer. The presence of storage lipids in nearly all prokaryotes is evidence for their strong evolutionary advantage. Indeed, the few prokaryotes lacking lipid accumulation exist in nutrient-rich habitats, where they would be expected to benefit little from energy storage [3].

Critical to the evolution of lipid-based energy storage was the development of neutral lipid synthesis within an endoplasmic reticulum, the emergence of lipid droplets as dedicated storage organelles, and the emergence of intercellular lipid trafficking mechanisms. Neutral lipid synthesis likely originated in actinomycetes and has persisted in eukaryotes as both an energy source and a safe haven for toxic metabolites [4-6]. Lipid droplets emerge from the endoplasmic reticulum and perform multiple critical functions: they sequester neutral lipids away from other cellular compartments, preventing lipotoxicity, and maintain spatial segregation of the lipid storage pool [7-9]. Lipid droplets also enable regulated energy mobilization through controlled lipolysis coupled with lipid trafficking to mitochondria. Lipid trafficking extends beyond individual cells in multicellular organisms, where large lipid transfer proteins (LLTPs) allow for multicellular control of energy metabolism [10]. Recent Cryo-EM structure of native honey bee vitellogenin links the emergence of LLTPs to multicellularity and to the evolutionary advantage of lipid transport mechanisms in the regulation of systemic energy needs [11].

## 3. Evolution of adipose tissue

A further refinement of lipid-based whole-body energy metabolism was the evolution of adipocytes, cells equipped with specialized lipid droplets that enable coordinated storage and mobilization of lipids to meet whole-organism energy demands. Adipocytes appear to have evolved early in vertebrate history, prior to the divergence of cartilaginous and bony fish approximately 450 million years ago [12, 13]. Cartilaginous fish such as sharks subsequently evolved to rely primarily on the liver for lipid storage [14], while bony fish retained specialized adipose tissue with conserved adipogenic machinery [15]. The presence of adipocytes across diverse bony fish lineages, combined with the teleost-specific 3R whole genome duplication [16, 17], may have contributed to the massive adaptive radiation of teleost fish.

Distributing lipid storage beyond the liver confers advantages by reducing hepatocellular stress, increasing metabolic plasticity, decentralizing fuel reserves, and enabling localized metabolic control across tissues [18]. While the presence of adipocytes is not sufficient, as non-teleost ray-finned fishes possess adipocytes yet remain species-poor [12, 13], the advantage of adipocyte-based lipid storage in the context of genome duplication may have been a key driver of diversification.

The evolution from simple chemiosmotic coupling to modern adipose tissue metabolism represents billions of years of refinement—through lipid synthesis, specialized lipid droplets, hormonal regulatory systems, and its ubiquitous distribution throughout the body, adipose tissue has evolved as the mastermind of the organism’s energy requirements. Nowhere is the critical dependency on adipocytes more apparent than in the severe metabolic consequences experienced by individuals with lipodystrophies, where absent or insufficient adipose tissue function leads to liver steatosis, severe insulin resistance, and all the cardiometabolic sequelae and mortality associated with metabolic disease [19-21].

## 4. Adipose tissue subtypes

In order to accommodate the energy storage and utilization needs of the whole organism, adipocytes have evolved to acquire functions other than lipid storage. These include the ability to sense organismal energy needs, to communicate the availability of stored energy, and to parse energy utilization in accordance with energy stores [22]. These functions require communication from and to the nervous system, optimization of fuel delivery to other tissues, and regulation of whole-body energy utilization. Consequently, adipocytes have the capacity to influence neurogenesis and angiogenesis and exert endocrine control of lipid utilization.

Further refinement is achieved by the presence of adipocytes in the close vicinity of organs, where their properties could directly affect cells composing those tissues. For example, specialized depots, such as mammary adipose tissue, contain adipocytes critical for lactation [23], while adipocytes within the bone marrow affect hematopoiesis and bone metabolism [24, 25]. Another example is adipocytes within the dermis, which contribute to immune responses, wound healing, hair follicle growth, and thermoregulation [26, 27]. Adipocytes are also present around the heart [28], surrounding major blood vessels [29], and in the perineurial region of nerves [30, 31]. Single-cell RNA sequencing studies have shown distinct adipocyte subtypes, which can be specialized for lipogenesis, mitochondrial oxidation, inflammatory signaling, or extracellular matrix remodeling [32, 33]. While we know relatively little about the role of adipocytes in every possible context, one of the specializations that has received much attention is the ability of adipocytes to maintain core temperature in the face of cold exposure by generating heat.

## 5. Thermogenic adipose tissue

In mammals, heat generation has been essential for survival in cold environments. Skeletal muscle contributes to maintaining body temperature through shivering thermogenesis, a process in which rapid, involuntary, asynchronous muscle contractions generate heat through extremely high rates of ATP turnover. During shivering, cross-bridge cycling between actin and myosin consumes ATP at accelerated rates, and each round of ATP hydrolysis liberates heat. In parallel, Ca^2+^ is repeatedly released and resequestered by the sarcoplasmic reticulum, and the SERCA pumps responsible for Ca^2+^ reuptake constitute an additional major ATP sink [34]. These combined processes cause a sharp increase in mitochondrial oxidative phosphorylation to meet ATP demand. Although heat production during shivering depends on chemiosmosis to generate ATP, heat itself is a secondary consequence of ATP hydrolysis and ion cycling, not the primary biological output of the mechanism.

A major evolutionary step was the emergence of non-shivering thermogenesis, a mechanism specifically designed to generate heat as its principal output [35]. This process is characteristic of brown adipocytes, which reside in highly vascularized depots (BAT) strategically positioned near vital organs. Brown adipocytes differ from “white” adipocytes in that they store triglycerides in smaller lipid droplets and have abundant mitochondria (hence the brown color) that can rapidly oxidize fatty acids derived from both their own lipid stores and neighboring white adipocytes [36]. Brown adipocyte mitochondria generate heat by leveraging the chemiosmotic gradient in a manner fundamentally distinct from muscle. Rather than fully coupling the proton-motive force to ATP synthesis, brown adipocytes express the mitochondrial innermembrane transporter UCP1, which, by enabling regulated proton conductance into the matrix, decreases the proton gradient [37]. By dissipating the proton gradient, UCP1 forces increased lipid oxidation and electron transport, effectively uncoupling substrate oxidation from ATP production. The chemical energy of increased electron transport is released directly as heat. By channeling an energy-dense fuel source—lipids—into sustained oxidation and electron transport, brown adipocytes generate heat as a primary biological output, without imposing mechanical or metabolic burdens on other tissues.

Unlike small mammals that heavily rely on brown fat for thermoregulation, humans possess alternative mechanisms to defend against cold environments, including behavioral adaptations (clothing and shelter), shivering thermogenesis, and vasomotor responses. For decades, it was believed that BAT involuted after infancy in humans and had no physiological significance in adults. However, landmark studies in 2009 [38-41] identified metabolically active BAT in adult humans using 18F-fluorodeoxyglucose positron emission tomography-computed tomography (18F-FDG PET-CT) imaging, sparking renewed interest in this tissue. A key observation emanating from these studies was that, in contrast to rodent brown adipocytes, which are found in a distinct, homogenous depot, human UCP1-expressing adipocytes were interspersed within pockets of larger unilocular white adipocytes. Moreover, these adipocytes are larger than canonical brown adipocytes and contain mitochondria in quantities higher than in white but lower than in brown adipocytes. This resulted in the terms brite (brown in white) and beige (in between brown and white) to describe these cells [42-44]. Subsequent studies identified beige adipocytes in the subcutaneous adipose tissue of rodents, and assigned distinct genetic signatures and developmental mechanisms to these cells [43, 45, 46].

The enthusiasm for studying thermogenic adipose tissue in humans intensified considerably due to striking epidemiological evidence linking its presence and activity to improved metabolic health. Large-scale retrospective analyses of PET-CT scans from over 52,000 patients revealed that individuals with detectable BAT had significantly lower prevalence of cardiometabolic diseases [47]. Critically, the protective associations between BAT and reduced risk of type 2 diabetes, dyslipidemia, coronary artery disease, cerebrovascular disease, congestive heart failure, and hypertension were independent of body mass index (BMI) and total adiposity. These findings have raised the possibility of thermogenic adipose tissue as a target for combating metabolic diseases, even when its thermogenic function may be less critical for survival than in other mammals.

A crucial question in this context is whether the association between thermogenic adipose tissue abundance and lower cardiometabolic disease burden is causal, i.e., do thermogenic adipocytes directly cause amelioration of metabolic disease, or do they emerge in response to coinciding protective factors such as physical activity, cold exposure, dietary patterns, or genetic predisposition? It is also plausible that there is a bilateral relationship where brown adipose tissue causally promotes improved metabolism and is in turn responsive to protective factors. Several potential molecular mechanisms could underlie a causal relationship between thermogenic adipose tissue and cardiometabolic protection. As noted above, adipocytes powerfully regulate neural and vascular development and systemic metabolism via endocrine mechanisms. Thus, brown adipocytes may exert systemic effects on insulin sensitivity and metabolism in distant tissues through secretion of specific factors.

Studies to explore the potentially causal role of BAT in systemic metabolism have been predominantly conducted in mouse models. These studies include genetic manipulations to eliminate genes of interest specifically in brown or beige adipocytes through the use of UCP1 promoter-driven transgenesis, or floxed allele knockouts using UCP1 promoter-driven Cre recombinase. While subject to potentially unaccounted for variables [48, 49], many of these studies support a role for BAT in the control of systemic metabolism [50-53]. Studies in which brown adipose tissue abundance is acutely increased through transplantation have also provided insights into its function. Stanford et al. first transplanted BAT from male donor mice into the visceral cavity of age- and sex-matched recipient mice, and demonstrated improved glucose tolerance, increased insulin sensitivity, lower body weight, decreased fat mass, and a reversal of high-fat diet-induced insulin resistance in transplanted mice [54]. Multiple additional studies since then have shown metabolic improvements upon transplantation of brown fat in normal, obese, and insulin-resistant models [55-60].

Beige adipocytes also appear to confer a positive metabolic benefit. The formation of beige adipocytes in mice depends on the ubiquitous transcriptional regulator PRDM16. Specific ablation of PRDM16 using UCP1-diven Cre recombinase results in the loss of beige adipocytes from the subcutaneous adipose tissue of mice following cold exposure. The mice developed obesity on a high-fat diet, with severe insulin resistance and hepatic steatosis [43]. The beneficial effect of beige fat is also evidenced by transplantation experiments. For example, Park et al. [61] harvested subcutaneous adipose tissue from mice expressing VEGF-A, which induces beiging, into diet-induced obese mice, and observed systemic metabolic benefits and reduced inflammation. Blumenfeld et al. [62] conducted in vitro beiging of subcutaneous adipose tissue, and observed enhanced weight loss of obese mice after cold exposure. Other transplantation studies confirm the observed metabolic benefits and also find antiinflammatory and neuroprotective effects of transplanted beige adipose tissue [63]. Moreover, additional studies reviewed previously find that beige fat improves fat grafting, potentially through enhanced angiogenesis [58, 64]. Translational studies in which human adipocytes are engrafted into immunocompromised mice also find improvements in weight, glucose tolerance, and hepatic steatosis [65, 66].

## 6. Mechanisms underlying the function of thermogenic adipose tissue

Studies directed towards identifying mechanisms underlying the beneficial effects of BAT and beige adipocytes have provided evidence for BAT-enriched factors, including lipids and metabolites [67-69]. These include Fibroblast Growth Factor 21 (FGF21), which is released under thermogenic activation and affects liver, heart, and improves insulin sensitivity; Neuregulin 4 (NRG4), which reduces hepatic steatosis and improves insulin sensitivity [70]; SLIT2, which is secreted by beige adipocytes and is in human plasma negatively correlated with plasma glucose levels [71, 72]; s100b, which stimulates innervation [73], and others [67-69]. Bioactive lipids have also been shown to be secreted by brown adipose tissue, including 12,13-dihydroxy-9Z-octadecenoic acid (12,13-diHOME) [74, 75]. Many more factors have been identified in un-biased screening studies [40, 76], including hormones, growth factors, ECM proteins, and complement factors.

**Figure 2 •:**
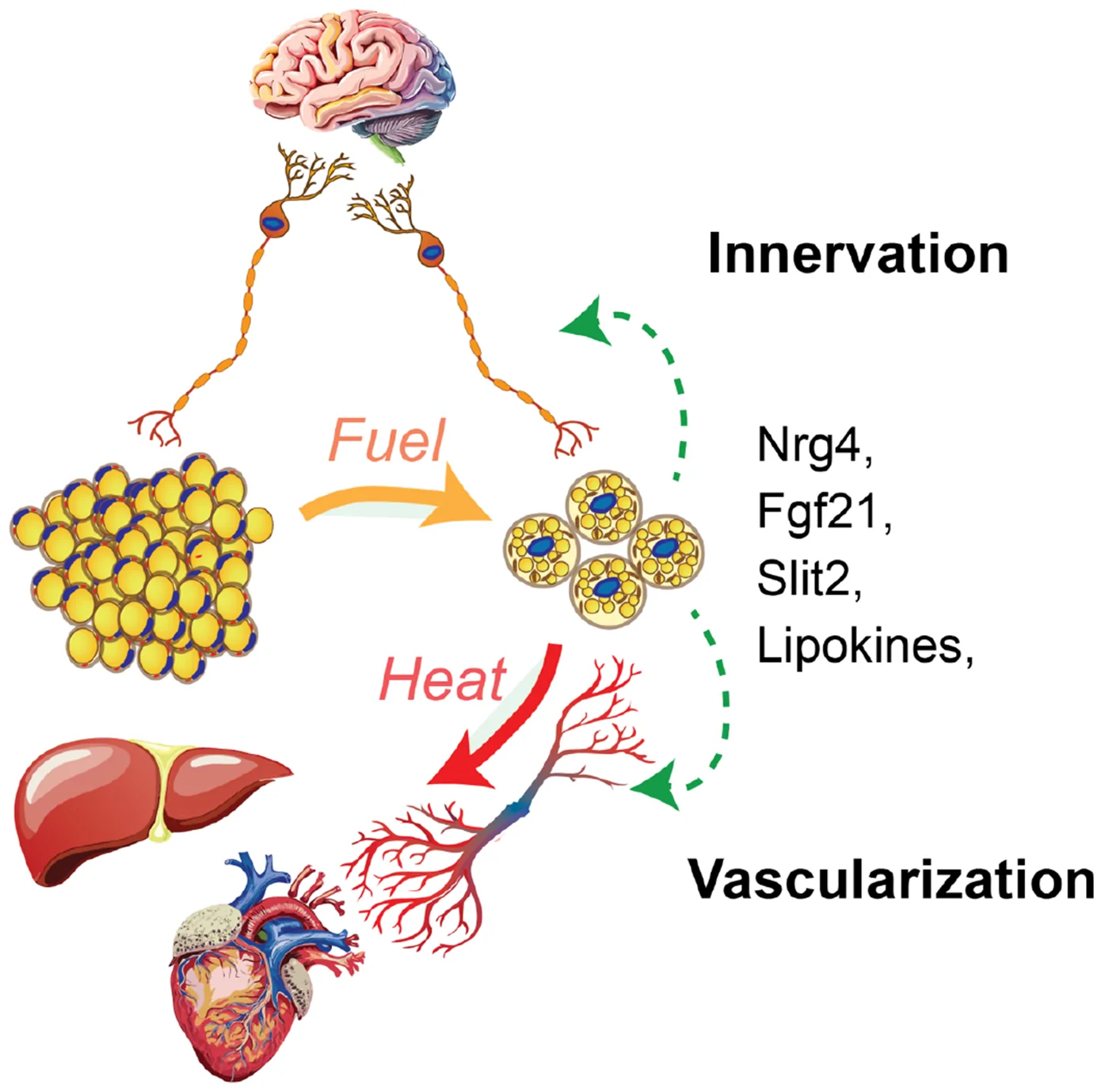
Thermogenic adipocytes orchestrate systemic fuel mobilization and heat dissipation. Sympathetic innervation provides rapid regulatory control of thermogenic adipocytes, promoting mobilization of lipid substrates from peripheral depots to fuel mitochondrial uncoupling and heat generation in brown and beige adipose tissue. In parallel, thermogenic adipocytes secrete peptide and lipid mediators, including Nrg4, Fgf21, Slit2, and lipokines, which modulate vascular remodeling and neural plasticity, reinforcing tissue perfusion and innervation. Increased vascularization supports both substrate delivery and heat dissipation, enabling thermogenic adipose tissue to influence whole-body physiological adaptation.

Why do brown and beige adipocytes express numerous factors that are not ostensibly required for cellular substrate oxidation or heat generation via mitochondrial uncoupling? While heat production through mitochondrial uncoupling is cell-autonomous, physiologically relevant thermogenesis is fundamentally a noncell-autonomous process, requiring coordinated systemic responses (Figure 2). These responses include the mobilization of lipids from storage sites for delivery to BAT, delivery of substrates to thermogenic adipocytes within the tissue, and efficient targeting of these substrates to mitochondria within the adipocytes. Heat generated by brown and beige adipocytes must be dissipated to core organs while minimizing peripheral heat loss. These processes require robust responsiveness of storage depots to mobilization signals, requiring neural and vascular supplies, and efficient distribution of generated heat via specific cardiovascular adaptations.

The secretion of signaling molecules from brown and beige adipocytes is likely required to orchestrate the distal response network necessary for organismal defense against cold exposure, and the wide-ranging metabolic benefits of brown and beige adipose tissue activation may be an ancillary result of these mechanisms. From this perspective, the possibility of harnessing the metabolic benefits of brown and beige adipocytes for therapeutic use will require understanding brown and beige adipocytes not as isolated thermogenic units, but as central nodes in a systemic network that serves to coordinate the organismal defense against cold exposure, with metabolic health benefits emerging as consequences of this coordination.

## 7. Control of the brown and beige adipocyte thermogenic programs

What are the stimuli that regulate abundance and activity of brown and beige adipocytes? As expected, given their role in thermogenesis, cold exposure is the strongest stimulus bringing about an increase in these cells [77]. In mice, acute exposure to cold induces expression of UCP1 in inguinal beige adipocytes, which can cycle between an activated, UCP1-expressing phenotype and a white, UCP1-repressed phenotype (dormant beige adipocytes) [78]. Chronic exposure to cold leads to increased volume of interscapular brown adipose tissue. In humans, acute exposure to cold activates glucose uptake in supraclavicular, perirenal, and paravertebral regions [79], and chronic exposure leads to enhancement in glucose uptake, consistent with an increase in the abundance of thermogenic adipocytes. Cold exposure causes activation of the sympathetic nervous system, leading to release of norepinephrine and activation of beta-adrenergic receptors in adipocytes. The response to the consequent elevation of cAMP and activation of protein kinase A varies depending on the adipocyte subtype. In white adipocytes, elevation of cAMP leads to lipolysis, while in UCP-1-expressing adipocytes, it leads to induction of UCP1 expression and activation of UCP1 uncoupling function, with consequent activation of fuel consumption and generation of heat [80]. In addition to cAMP-mediated mechanisms, increased UCP1 and brown/beige adipocyte abundance are seen in response to PPARγ activation by thiazolidinediones [81, 82]. Physiologically, this mechanism may be induced by endogenous PPARγ ligands released through lipolysis, thus coordinating cold-induced lipolysis to UCP1 induction [83].

White and beige adipocyte functions are governed by factors that activate transcription of genes in a cell-specific fashion. A higher-level control over these transcription factors is mediated by co-activators and co-repressors, which coordinate the activation or repression of multiple transcription factors at a time to achieve a complex response. Co-activators and co-repressors orchestrate transcriptional pathways that not only affect the cell autonomously but also affect its interactions with its environment. The co-activator PCG1α and the co-repressor NRIP1 are mediators of thermogenic adipocyte identity.

PGC1α was originally identified in mouse adipocytes as a potent co-activator of PPARγ, enhancing transcription of genes required for triglyceride synthesis, lipid-droplet formation, and terminal adipocyte identity. Mechanistically, PGC1α also co-activates NRF1/2 and ERRα/β/γ, driving expression of mitochondrial transcription factor A (TFAM), respiratory-chain proteins, and enzymes of β-oxidation [84-86]. This coordinated activation couples adipocyte differentiation to a parallel expansion of mitochondrial mass and oxidative capacity. In thermogenic adipocytes, PGC1α facilitates PPARγ-dependent induction of UCP1, enabling dissipation of the proton-motive force and diverting substrate oxidation toward heat production rather than ATP synthesis. In this way, PGC1α organizes a multi-layered program—lipid mobilization, mitochondrial influx of fatty acids, β-oxidation, respiratory uncoupling—that collectively establishes the thermogenic cellular machinery.

Conversely, NRIP1 (also known as RIP140) represses many of the functions that are co-activated by PGC1alpha. This co-repressor was first identified as an interacting protein with the estrogen receptor [87], and subsequently found to play a key role in regulating systemic energy balance; mice lacking NRIP1 are resistant to obesity and hepatic steatosis, consistent with a heightened capacity for whole-body oxidative nutrient disposal [88]. Further studies found that NRIP1 suppresses UCP1 expression in adipocytes [89, 90], which, at least in part, is likely to mediate its systemic metabolic effects.

The systemic influence of thermogenic adipocytes is further supported by the remarkable finding that implantation of either mouse or human adipocytes lacking NRIP1 into mice can mitigate obesity, glucose intolerance, and hepatic steatosis [65]. These experiments demonstrate that a relatively small volume of adipose tissue reprogrammed to enhance thermogenesis can exert systemwide metabolic benefits, supporting the view that thermogenic adipocytes function as a central node in the systems that orchestrate fuel utilization. The fact that this effect is observed using both mouse and human adipocytes underscores the conservation of these mechanisms and highlights the potential translational relevance. Importantly, these implant models provide a uniquely tractable platform to dissect the endocrine signals that coordinate systemic fuel partitioning.

## 8. Adipose tissue transformations during development and aging

**Figure 3 •:**
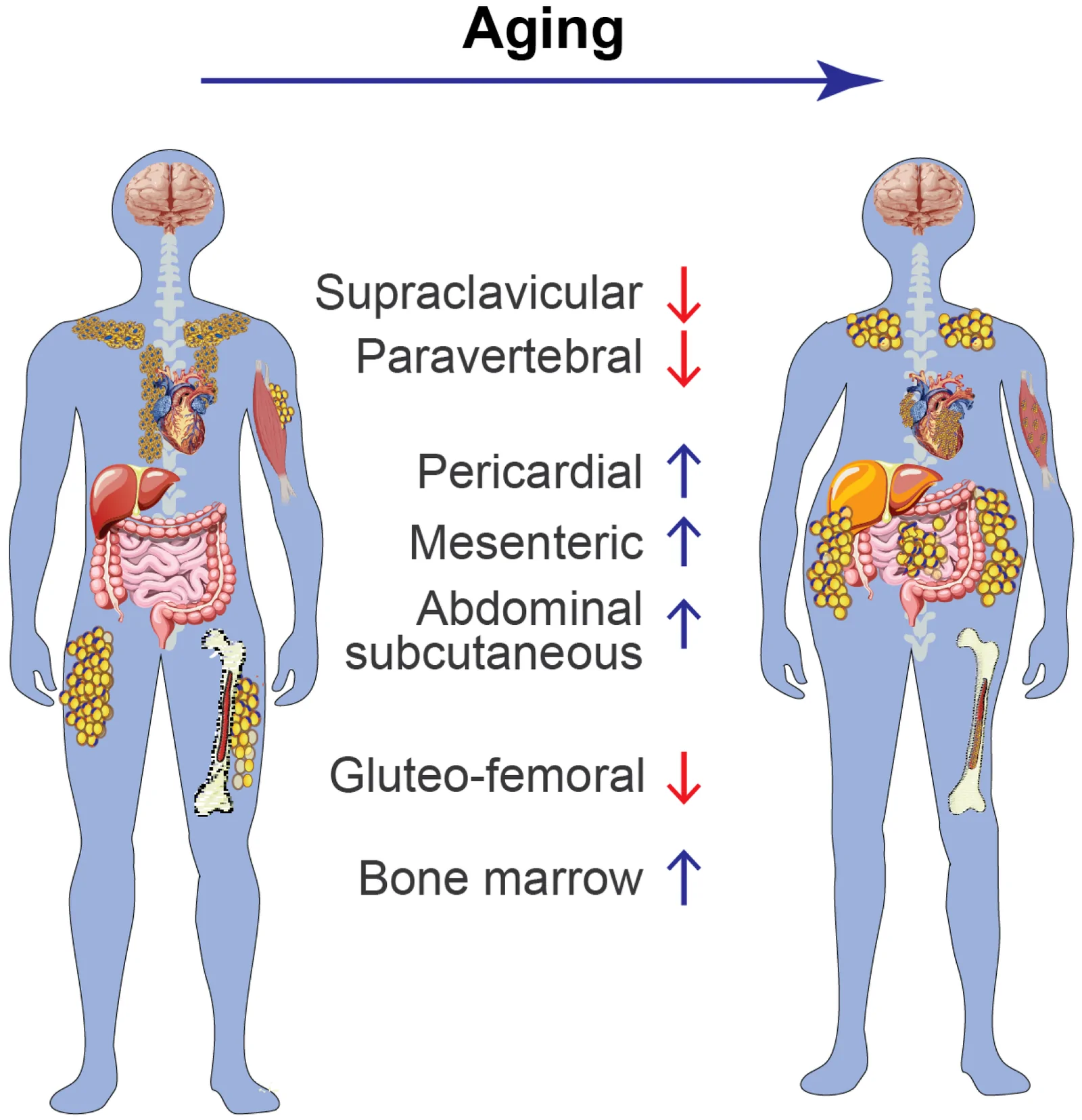
Age-associated redistribution of adipose depots. Upper-body depots, including supraclavicular and paravertebral beige adipose tissue, decline with age (red arrows), whereas pericardial, mesenteric, abdominal subcutaneous, and bone marrow adipose tissue increase (blue arrows). In contrast, gluteo-femoral subcutaneous adipose tissue decreases. Arrows indicate the direction of change rather than absolute mass. The figure highlights redistribution of lipid storage across anatomical sites with aging and does not imply uniform mechanisms or rates of change across depots.

Adipose tissue forms in utero, with thermogenic adipose tissue being prevalent in newborn babies [91]. Over the lifetime, there are major changes in adipose tissue distribution, coinciding with developmental phases including puberty and pregnancy. The transformations in adipose tissue continue post-reproductively through the aging process. The major changes occurring with aging are increases in BMI [92], the redistribution of adipose tissue that lowers subcutaneous depots and increases central depots [93, 94], the disappearance of thermogenic adipose tissue [95], and the accumulation of adipocytes in the bone marrow [96] (Figure 3). While an increased risk of metabolic disease is seen with normal-to-overweight trajectories in body mass index with aging [92], a significant increase in type 2 diabetes risk occurs independently of body mass index [97]. These results suggest that other changes in adipose tissue may contribute more strongly to risk. This possibility is supported by observations that, at any age, the distribution of adipose tissue between the subcutaneous and central depots and the abundance of thermogenic adipose tissue impact metabolic disease risk. These observations support the notion that qualitative depot remodeling may be more consequential than BMI alone.

## 9. How do these developmental transformations occur?

A remarkable feature of adipocytes is that they are exceptionally long-lived. Spalding et al. used atmospheric ^14^C generated by mid-20th-century nuclear testing as a natural tracer to measure adipocyte turnover in adults, and found that only ~10% of fat cells are renewed annually—a rate that remains consistently slow across adult ages and BMI categories [98]. These findings imply that the shifts in adipose tissue distribution observed later in life are not the product of rapid remodeling, but instead reflect developmental trajectories initiated decades earlier. Supporting this idea, similar age-related redistribution patterns—including increased visceral adiposity—are observed across multiple species [99], while thermogenic adipose tissue progressively diminishes with age [100].

In this context, understanding how adipocytes and their distinct subtypes are generated in specific depots becomes crucial for identifying strategies to mitigate cardiometabolic risk during aging. Because mature adipocytes are post-mitotic, the formation of new adipocytes depends entirely on the recruitment and differentiation of progenitor cells. These mesenchymal progenitors reside within adipose depots and are exquisitely sensitive to systemic cues. For example, parabiosis experiments linking young and old animals show that progenitor cells in adipose tissue respond robustly to circulating factors from the partner animal, highlighting their responsiveness to systemic age-related signals [101].

Despite their crucial role in determining systemic metabolic health, the in vivo mechanisms governing progenitor proliferation, activation, and depot-specific behavior are slowly being understood [99, 102, 103] but remain largely unknown. It is plausible that these progenitors operate under conserved developmental signaling pathways, including Wnt signaling, which has been implicated in lineage specification; regional adipose identity; and the balance between white, beige, and brown adipocyte fates [104-107]. Understanding how these pathways—shaped over millions of years to control energy storage and thermogenesis—continue to operate across the lifespan may ultimately explain why adipose tissue remodeling with age so powerfully affects cardiometabolic health.

## 10. What can we do with what we know?

Although our understanding of adipose tissue biology remains fragmentary, the findings summarized above point to potential translational possibilities. The identification of powerful regulatory pathways such as NRIP1 and the observation that its inhibition can shift adipocytes toward a more oxidative and thermogenic state suggest that relatively modest perturbations can have meaningful systemic effects. Strategies based on siRNA-mediated NRIP1 knockdown could potentially generate metabolically improved adipocytes with greater capacity to integrate systemic metabolism. More broadly, white adipocytes can be steered toward beneficial phenotypes through targeted interventions, highlighting their inherent plasticity. In parallel, factors secreted by thermogenic or reprogrammed adipocytes, including FGF21 and many others not yet characterized, may provide additional therapeutic entry points [108, 109]. Even with the limited knowledge we currently have, these examples illustrate that manipulating adipocyte regulatory networks, their endocrine outputs, or the progenitor cells that give rise to them could offer new approaches to improving metabolic health.

## Data Availability

No new data were created or analyzed in this study. Data sharing is not applicable to this article.
